# Supplementary material for: The role of the nuclear pore complex in the stability of disease-related short tandem DNA repeats
Source: Nucleic Acids Res. 2026 May 20;54(10):gkag496. doi: 10.1093/nar/gkag496 (PMC13187838; doi:10.1093/nar/gkag496)
Supplement: gkag496_Supplemental_File [file gkag496_supplemental_file.pdf]

## Supplemental material

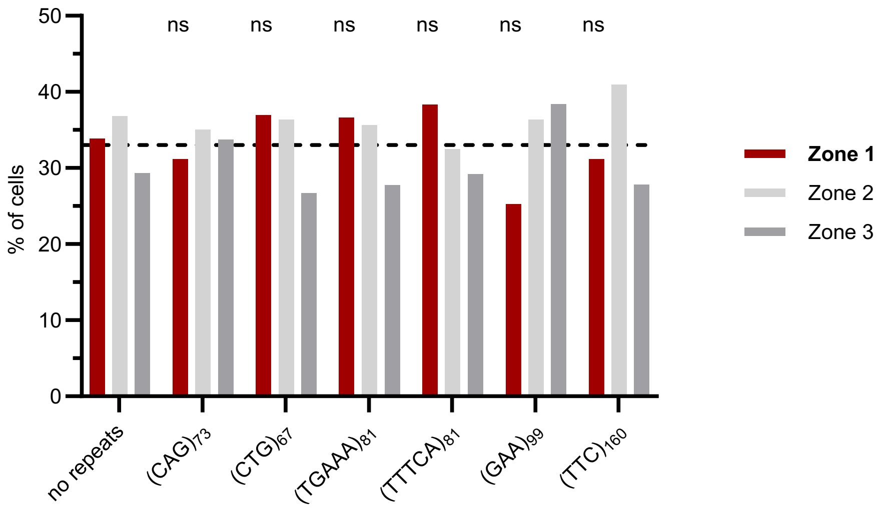


Figure S1. Subnuclear localization of the indicated STRs in G1 phase cells, defined by the absence of a bud, as determined by the zoning assay (complement to Fig. 2D). ns: not significant compared to cells without STRs inserted (zone 1) by Fisher's exact test.

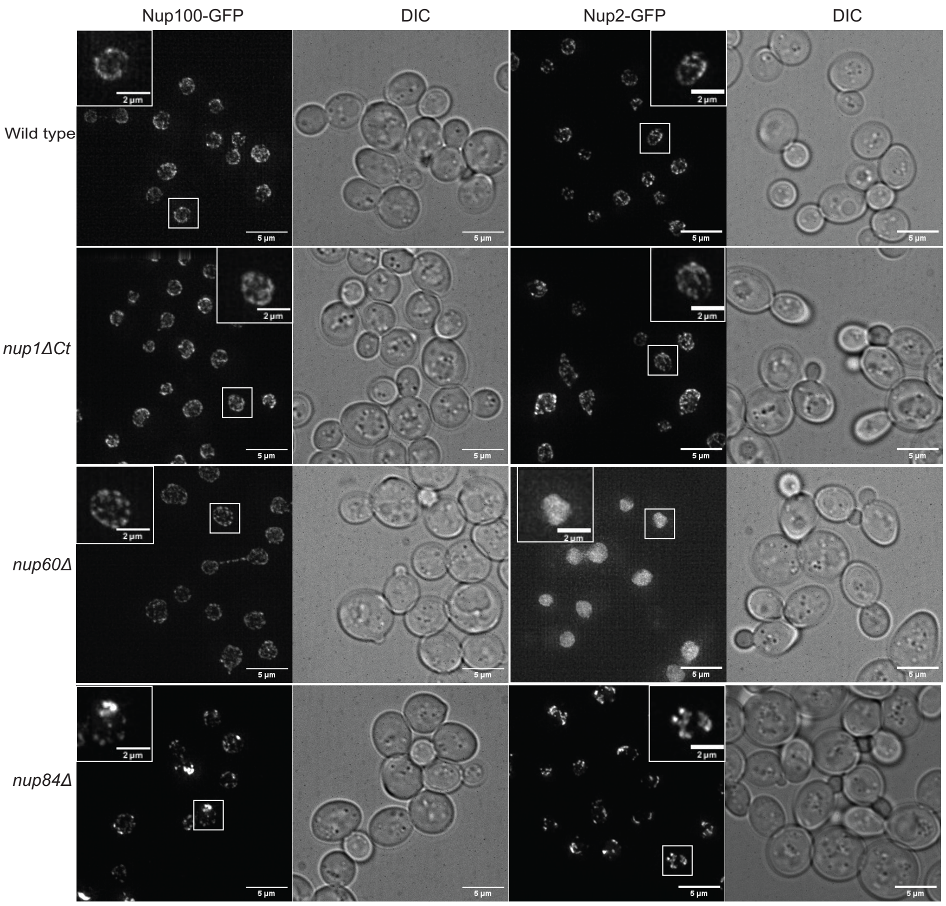


Figure S2. Representative fluorescence microscopy images of endogenously expressed Nup100-GFP and Nup2-GFP in strains of the indicated genotypes. Images represent maximum intensity projections; scale bar: 5 µm (inset, 2 µm)

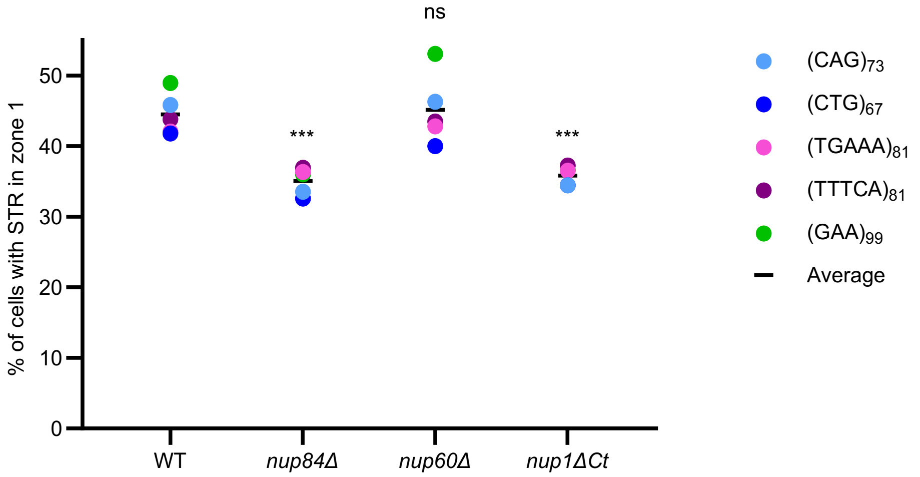


Figure S3. Percentage of cells in S/G2 phase with GFP signal in zone 1. Different representation of data from Fig. 3C, with average percentage of all STRs indicated. p-values were calculating using a pooled mixed-effects logistic regression with dataset included as a random effect. *nup84Δ*: odds ratio (OR) = 0.674, 95% confidence interval (CI) [0.551, 0.823], p = 0.0001; *nup60Δ*: OR = 1.018, 95% CI [0.842, 1.230], p = 0.857, *nup1ΔCt*: OR = 0.696, 95% CI [0.575, 0.842], p = 0.0002.

Table S1. Plasmids used in this study.

| Name | Description | Integration site | Source |
|---|---|---|---|
| pGC542 | Vector for STR cloning and expansion (PacI-NotI sites) | - | (Casas-Delucchi et al., 2022) |
| pDN46.1 | pGC542 + PacI-BsaI-$(CTG)_{81}$-BsmBI-NotI | - | This study |
| pDN47.4 | pGC542 + PacI-BsaI-$(CTG)_{101}$-BsmBI-NotI | - | This study |
| pDN48.1 | pGC542 + PacI-BsaI-$(CTG)_{121}$-BsmBI-NotI | - | This study |
| pSW03 | pGC542 + PacI-BsaI-$(CTG)_{161}$-BsmBI-NotI | - | (Casas-Delucchi et al., 2022) |
| pDN72.1 | pGC542 + PacI-BsaI-$(TGAAA)_{41}$-BsmBI-NotI | - | This study |
| pDN74.1 | pGC542 + PacI-BsaI-$(TGAAA)_{51}$-BsmBI-NotI | - | This study |
| pDN75.2 | pGC542 + PacI-BsaI-$(TGAAA)_{61}$-BsmBI-NotI | - | This study |
| pDN76.1 | pGC542 + PacI-BsaI-$(TGAAA)_{81}$-BsmBI-NotI | - | This study |
| pDN109.2 | pGC542 + PacI-BsaI-$(TGAAA)_{161}$-BsmBI-NotI | - | This study |
| pDN33.2 | pGC542 + PacI-BsaI-$(GAA)_{81}$-BsmBI-NotI | - | This study |
| pDN51.3 | pGC542 + PacI-BsaI-$(GAA)_{160}$-BsmBI-NotI | - | This study |
| pAG32 | pAgTEF1-hphMX tAgTEF1 | - | (Goldstein and McCusker, 1999) |
| pDN7.8 | Vector for repeats integration for GCR assay<br>SalI - PRB1 homology UP - HphMX - PacI - NotI - PRB1 homology DOWN – SalI | *PRB1* | This study |
| pDN12.1 | Vector for repeats integration for GCR assay<br>SalI - PRB1 homology UP - HphMX - NotI - PacI - PRB1 homology DOWN – SalI | *PRB1* | This study |
| pDN53.1 | pDN12.1 + NotI- BsmBI-$(CAG)_{101}$- BsaI-PacI | *PRB1* | This study |
| pDN54.1 | pDN12.1 + NotI- BsmBI-$(CAG)_{121}$- BsaI-PacI | *PRB1* | This study |
| pDN13.10 | pDN12.1 + NotI- BsmBI-$(CAG)_{161}$- BsaI-PacI | *PRB1* | This study |
| pDN133.1 | pDN7.8 + PacI-BsaI-$(CTG)_{81}$-BsmBI-NotI | *PRB1* | This study |
| pDN103.2 | pDN7.8 + PacI-BsaI-$(TGAAA)_{41}$-BsmBI-NotI | *PRB1* | This study |
| pDN85.1 | pDN7.8 + PacI-BsaI-$(TGAAA)_{51}$-BsmBI-NotI | *PRB1* | This study |
| pDN87.1 | pDN7.8 + PacI-BsaI-$(TGAAA)_{61}$-BsmBI-NotI | *PRB1* | This study |
| pDN89.1 | pDN7.8 + PacI-BsaI-$(TGAAA)_{81}$-BsmBI-NotI | *PRB1* | This study |
| pDN113.2 | pDN7.8 + PacI-BsaI-$(TGAAA)_{161}$-BsmBI-NotI | *PRB1* | This study |
| pDN104.1 | pDN12.1 + NotI- BsmBI-$(TTTCA)_{41}$- BsaI-PacI | *PRB1* | This study |
| pDN86.1 | pDN12.1 + NotI- BsmBI-$(TTTCA)_{51}$- BsaI-PacI | *PRB1* | This study |
| pDN88.2 | pDN12.1 + NotI- BsmBI-$(TTTCA)_{61}$- BsaI-PacI | *PRB1* | This study |
| pDN90.2 | pDN12.1 + NotI- BsmBI-$(TTTCA)_{81}$- BsaI-PacI | *PRB1* | This study |
| pDN114.4 | pDN12.1 + NotI- BsmBI-$(TTTCA)_{161}$- BsaI-PacI | *PRB1* | This study |
| pDN40.3 | pDN7.8 + PacI-BsaI-$(GAA)_{81}$-BsmBI-NotI | *PRB1* | This study |
| pDN58.1 | pDN7.8 + PacI-BsaI-$(GAA)_{160}$-BsmBI-NotI | *PRB1* | This study |
| pDN41.1 | pDN12.1 + NotI- BsmBI-$(TTC)_{81}$- BsaI-PacI | *PRB1* | This study |
| pDN59.1 | pDN12.1 + NotI- BsmBI-$(TTC)_{160}$- BsaI-PacI | *PRB1* | This study |
| pADH4UCA | EcoRI-URA3 marked telomere 7L-SalI | - | (de Bruin et al., 2001) |
| pSO31 | Vector for repeats integration for zoning assay<br>SalI – HIS2 homology UP - URA3 - PacI - NotI - tRNA homology DOWN - SalI | *HIS2* | This study |
| pSO32 | Vector for repeats integration for zoning assay<br>SalI – HIS2 homology UP - URA3 - NotI - PacI - tRNA homology DOWN - SalI | *HIS2* | This study |
| pSO20 | pADH4UCA + BamH1-$(CAG)_{70}$ -EcoRI | *HIS2* | This study |
| pSO24 | pADH4UCA + BamH1- $(CTG)_{67}$-EcoRI | *HIS2* | This study |
| pDN91.1 | pSO31 + PacI-BsaI-$(GAA)_{160}$-BsmBI-NotI | *HIS2* | This study |

| | | | |
|---|---|---|---|
| pDN101.1 | pSO31 + PacI-BsaI-$(TGAAA)_{81}$-BsmBI-NotI | *HIS2* | This study |
| pDN102.1 | pSO32 + PacI-BsaI-$(TTTCA)_{81}$-BsmBI-NotI | *HIS2* | This study |
| pISL-URA-TRP1-ISR | pYES3 -UR-$(GAA)_{100}$-Tet269-A3 - TRP1 | ARS306 | (Aksenova et al., 2013) |
| pSO82 | Vector for repeats integration for contraction assay BaeI - ARS306 homology UP pYES3-UR-PacI-NotI-A3 - TRP1 - ARS306 homology DOWN - EcoRI | ARS306 | This study |
| pSO85 | BaeI - ARS306 homology UP pYES3 -UR-PacI-BsaI-$(GAA)_{121}$-BsmBI-NotI-A3 - TRP1 - ARS306 homology DOWN - EcoRI | ARS306 | This study |
| pSO53 | pPGK1-Cas9-tPGK1 - NUP133-sgRNA – NatMX | - | This study |

Table S2. Reagents used in this study.

| Reagent | Source | Identifier |
|---|---|---|
| Synthetic DNA fragments | Integrated DNA Technologies | |
| *PCR* | | |
| Deoxynucleotide (dNTP) Solution Set | New England Biolabs | N0446S |
| Q5® High-Fidelity DNA Polymerase | New England Biolabs | M0491L |
| OneTaq® 2X Master Mix with Standard Buffer | New England Biolabs | M0482 |
| *SD medium* | | |
| D-glucose anhydrous (20 g/L) | Fisher Chemical | 10141520 |
| Yeast nitrogen base without amino acids (6.7 g/L) | Becton Dickinson | 291920 |
| Adenine sulfate (43 mg/L) | Acros Organics | 163631000 |
| L-arginine (21 mg/L) | Acros Organics | 105001000 |
| L-aspartic acid (103 mg/L) | Thermo Scientific | 105045000 |
| L-glutamic acid (103 mg/L) | Alfa Aesar | A12919 |
| L-histidine (21 mg/L) | Acros Organics | 166151000 |
| L-leucine (62 mg/L) | Acros Organics | 125121000 |
| L-lysine (31 mg/mL) | Acros Organics | 125221000 |
| L-methionine (21 mg/L) | Acros Organics | 166161000 |
| L-phenylalanine (52 mg/L) | Acros Organics | 130311000 |
| L-serine (388 mg/L) | Acros Organics | 1322665000 |
| L-threonine (207 mg/L) | Fisher Scientific | BP394-100 |
| L-tryptophan (41 mg/L) | Thermo Scientific | 140591000 |
| L-tyrosine (31 mg/L) | Acros Organics | 140641000 |
| L-valine (155 mg/L) | Thermo Scientific | 140811000 |
| Uracil (21 mg/L) | Sigma-Aldrich | U0750 |
| *YPD medium* | | |
| D-glucose anhydrous (20 g/L) | Fisher Chemical | 10141520 |
| Peptone | Becton Dickinson | 211677 |
| Yeast extract | Becton Dickinson | 212750 |
| Select Agar | Invitrogen | 30391-049 |
| *Antibiotics* | | |
| 5-Fluoroorotic acid | Thermo Fisher Scientific | R0812 |
| Ampicillin | Sigma-Aldrich | A9518 |
| Canavanine | Sigma-Aldrich | C9758 |
| Geneticin, G418 | Santa Cruz Biotechnology | sc-29065A |
| 1,6-Hexandiol | Sigma-Aldrich | 240117-50G |
| 2,5-Hexandiol | Sigma-Aldrich | H11904-50G |
| Concanavalin A | Sigma-Aldrich | L7647 |

Table S3. Yeast strains used in this study.

| Name | Genotype | Source |
|---|---|---|
| DNY222 | *MATα ADE2 CAN1 his3-11,15 leu2-3,112 lys2Δ trp1-1 ura3-1 RAD5 hxt13ΔURA3 prb1ΔhphMX* | This study |
| DNY176 | *MATα ADE2 CAN1 his3-11,15 leu2-3,112 lys2Δ trp1-1 ura3-1 RAD5 hxt13ΔURA3 prb1ΔhphMX-(CAG)$_{101}$* | This study |
| DNY182 | *MATα ADE2 CAN1 his3-11,15 leu2-3,112 lys2Δ trp1-1 ura3-1 RAD5 hxt13ΔURA3 prb1ΔhphMX-(CAG)$_{121}$* | This study |
| DNY144 | *MATα ADE2 CAN1 his3-11,15 leu2-3,112 lys2Δ trp1-1 ura3-1 RAD5 hxt13ΔURA3 prb1ΔhphMX-(CAG)$_{161}$* | This study |
| DNY244 | *MATα ADE2 CAN1 his3-11,15 leu2-3,112 lys2Δ trp1-1 ura3-1 RAD5 hxt13ΔURA3 prb1ΔhphMX-(CTG)$_{81}$* | This study |
| DNY188 | *MATα ADE2 CAN1 his3-11,15 leu2-3,112 lys2Δ trp1-1 ura3-1 RAD5 hxt13ΔURA3 prb1ΔhphMX-(GAA)$_{160}$* | This study |
| DNY189 | *MATα ADE2 CAN1 his3-11,15 leu2-3,112 lys2Δ trp1-1 ura3-1 RAD5 hxt13ΔURA3 prb1ΔhphMX-(TTC)$_{160}$* | This study |
| DNY203 | *MATα ADE2 CAN1 his3-11,15 leu2-3,112 lys2Δ trp1-1 ura3-1 RAD5 hxt13ΔURA3 prb1ΔhphMX-(TGAAA)$_{51}$* | This study |
| DNY205 | *MATα ADE2 CAN1 his3-11,15 leu2-3,112 lys2Δ trp1-1 ura3-1 RAD5 hxt13ΔURA3 prb1ΔhphMX-(TGAAA)$_{59}$* | This study |
| DNY207 | *MATα ADE2 CAN1 his3-11,15 leu2-3,112 lys2Δ trp1-1 ura3-1 RAD5 hxt13ΔURA3 prb1ΔhphMX-(TGAAA)$_{80}$* | This study |
| DNY234 | *MATα ADE2 CAN1 his3-11,15 leu2-3,112 lys2Δ trp1-1 ura3-1 RAD5 hxt13ΔURA3 prb1ΔhphMX-(TGAAA)$_{161}$* | This study |
| DNY204 | *MATα ADE2 CAN1 his3-11,15 leu2-3,112 lys2Δ trp1-1 ura3-1 RAD5 hxt13ΔURA3 prb1ΔhphMX-(TTTCA)$_{51}$* | This study |
| DNY206 | *MATα ADE2 CAN1 his3-11,15 leu2-3,112 lys2Δ trp1-1 ura3-1 RAD5 hxt13ΔURA3 prb1ΔhphMX-(TTTCA)$_{59}$* | This study |
| DNY208 | *MATα ADE2 CAN1 his3-11,15 leu2-3,112 lys2Δ trp1-1 ura3-1 RAD5 hxt13ΔURA3 prb1ΔhphMX-(TTTCA)$_{80}$* | This study |
| DNY235 | *MATα ADE2 CAN1 his3-11,15 leu2-3,112 lys2Δ trp1-1 ura3-1 RAD5 hxt13ΔURA3 prb1ΔhphMX-(TTTCA)$_{161}$* | This study |
| SOY138 | *MAT***a** *ADE2 his3-11,15 lys2Δ trp1-1 ura3-1 RAD5 leu2-3,112::LacI-GFP-LEU2 Chr6int::lacop-lexAop-TRP1 HMG1-mCherry-HygMX his2-tRNA::URA3* | This study |
| SOY125 | *MAT***a** *ADE2 his3-11,15 lys2Δ trp1-1 ura3-1 RAD5 leu2-3,112::LacI-GFP-LEU2 Chr6int::lacop-lexAop-TRP1 HMG1-mCherry-HygMX his2-tRNA::URA3-(CAG)$_{73}$* | This study |
| SOY127 | *MAT***a** *ADE2 his3-11,15 lys2Δ trp1-1 ura3-1 RAD5 leu2-3,112::LacI-GFP-LEU2 Chr6int::lacop-lexAop-TRP1 HMG1-mCherry-HygMX his2-tRNA:: URA3-(CTG)$_{67}$* | This study |
| SOY209 | *MAT***a** *ADE2 his3-11,15 lys2Δ trp1-1 ura3-1 RAD5 leu2-3,112::LacI-GFP-LEU2 Chr6int::lacop-lexAop-TRP1 HMG1-mCherry-HygMX his2-tRNA::URA3-(TTTCA)$_{81}$* | This study |
| SOY210 | *MAT***a** *ADE2 his3-11,15 lys2Δ trp1-1 ura3-1 RAD5 leu2-3,112::LacI-GFP-LEU2 Chr6int::lacop-lexAop-TRP1 HMG1-mCherry-HygMX his2-tRNA:: URA3-(TGAAA)$_{81}$* | This study |
| SOY212 | *MAT***a** *ADE2 his3-11,15 lys2Δ trp1-1 ura3-1 RAD5 leu2-3,112::LacI-GFP-LEU2 Chr6int::lacop-lexAop-TRP1 HMG1-mCherry-HygMX his2-tRNA:: URA3-(GAA)$_{99}$* | This study |
| SOY211 | *MAT***a** *ADE2 his3-11,15 lys2Δ trp1-1 ura3-1 RAD5 leu2-3,112::LacI-GFP-LEU2 Chr6int::lacop-lexAop-TRP1 HMG1-mCherry-HygMX his2-tRNA:: URA3-(TTC)$_{160}$* | This study |

| | | |
|---|---|---|
| SOY219 | *MAT**a** ADE2 his3-11,15 lys2Δ trp1-1 ura3-1 RAD5 leu2-3,112::LacI-GFP-LEU2 Chr6int::lacop-lexAop-TRP1 NUP159-mCherry-KanMX nup133ΔN his2-tRNA::URA3* | This study |
| SOY220 | *MAT**a** ADE2 his3-11,15 lys2Δ trp1-1 ura3-1 RAD5 leu2-3,112::LacI-GFP-LEU2 Chr6int::lacop-lexAop-TRP1 NUP159-mCherry-KanMX nup133ΔN his2-tRNA::*$(CAG)_{68}$*-URA3* | This study |
| SOY238 | *MAT**a** ADE2 his3-11,15 lys2Δ trp1-1 ura3-1 RAD5 leu2-3,112::LacI-GFP-LEU2 Chr6int::lacop-lexAop-TRP1 NUP159-mCherry-KanMX nup133ΔN his2-tRNA::*$(CTG)_{63}$*-URA3* | This study |
| SOY231 | *MAT**a** ADE2 his3-11,15 lys2Δ trp1-1 ura3-1 RAD5 leu2-3,112::LacI-GFP-LEU2 Chr6int::lacop-lexAop-TRP1 NUP159-mCherry-KanMX nup133ΔN his2-tRNA::*$(TGAAA)_{82}$*-URA3* | This study |
| SOY232 | *MAT**a** ADE2 his3-11,15 lys2Δ trp1-1 ura3-1 RAD5 leu2-3,112::LacI-GFP-LEU2 Chr6int::lacop-lexAop-TRP1 NUP159-mCherry-KanMX nup133ΔN his2-tRNA::*$(TTTCA)_{81}$*-URA3* | This study |
| SOY233 | *MAT**a** ADE2 his3-11,15 lys2Δ trp1-1 ura3-1 RAD5 leu2-3,112::LacI-GFP-LEU2 Chr6int::lacop-lexAop-TRP1 NUP159-mCherry-KanMX nup133ΔN his2-tRNA::*$(GAA)_{160}$*-URA3* | This study |
| SOY240 | *MAT**a** ADE2 his3-11,15 lys2Δ trp1-1 ura3-1 RAD5 leu2-3,112::LacI-GFP-LEU2 Chr6int::lacop-lexAop-TRP1 NUP159-mCherry-KanMX nup133ΔN his2-tRNA::*$(TTC)_{120}$*-URA3* | This study |
| MKY65 | *MATα ADE2 can1-100 leu2-3,112 his3-11,15 ura3-1 trp1-1 LYS2 RAD5 NUP1-GFP::HIS3MX6* | This study |
| MKY23 | *MAT**a** ade2 can1-100 leu2-3,112 his3-11,15 ura3-1 trp1-1 LYS2 RAD5 nup60Δ::KanMX NUP1-GFP::HIS3MX6* | This study |
| MKY26 | *MATα ADE2 can1-100 leu2-3,112 his3-11,15 ura3-1 trp1-1 LYS2 RAD5 nup84Δ::KanMX Nup1-GFP::HIS3MX6* | This study |
| MKY66 | *MATα ADE2 can1-100 leu2-3,112 his3-11,15 ura3-1 trp1-1 LYS2 RAD5 NUP60-GFP::HIS3MX6* | This study |
| MKY32 | *MATα ADE2 can1-100 leu2-3,112 his3-11,15 ura3-1 trp1-1 LYS2 RAD5 nup1ΔCt::KanMX NUP60-GFP::HIS3MX6* | This study |
| MKY36 | *MATα ADE2 can1-100 leu2-3,112 his3-11,15 ura3-1 trp1-1 LYS2 RAD5 nup84Δ::KanMX NUP60-GFP::HIS3MX6* | This study |
| MKY68 | *MATα ADE2 can1-100 leu2-3,112 his3-11,15 ura3-1 trp1-1 LYS2 RAD5 NUP84-GFP::HIS3MX6* | This study |
| MKY42 | *MATα ADE2 can1-100 leu2-3,112 his3-11,15 ura3-1 trp1-1 LYS2 RAD5 nup1ΔCt::KanMX NUP84-GFP::HIS3MX6* | This study |
| MKY44 | *MATα ADE2 can1-100 leu2-3,112 his3-11,15 ura3-1 trp1-1 LYS2 RAD5 nup60Δ::KanMX NUP84-GFP::HIS3MX6* | This study |
| SOY184 | *MAT**a** ADE2 his3-11,15 lys2Δ trp1-1 ura3-1 RAD5 leu2-3,112::LacI-GFP-LEU2 Chr6int::lacop-lexAop-TRP1 HMG1-mCherry-HygMX his2-tRNA::URA3 nup84ΔKanMX* | This study |
| SOY183 | *MAT**a** ADE2 his3-11,15 lys2Δ trp1-1 ura3-1 RAD5 leu2-3,112::LacI-GFP-LEU2 Chr6int::lacop-lexAop-TRP1 HMG1-mCherry-HygMX his2-tRNA::URA3 nup60ΔKanMX* | This study |
| SOY229 | *MAT**a** ADE2 his3-11,15 lys2Δ trp1-1 ura3-1 RAD5 leu2-3,112::LacI-GFP-LEU2 Chr6int::lacop-lexAop-TRP1 HMG1-mCherry-HygMX his2-tRNA::URA3 nup1ΔCt::KanMX* | This study |
| SOY143 | *MAT**a** ADE2 his3-11,15 lys2Δ trp1-1 ura3-1 RAD5 leu2-3,112::LacI-GFP-LEU2 Chr6int::lacop-lexAop-TRP1 HMG1-mCherry-HygMX his2-tRNA::URA3-*$(CAG)_{70}$ *nup84ΔKanMX* | This study |
| SOY128 | *MAT**a** ADE2 his3-11,15 lys2Δ trp1-1 ura3-1 RAD5 leu2-3,112::LacI-GFP-LEU2 Chr6int::lacop-lexAop-TRP1 HMG1-mCherry-HygMX his2-tRNA::URA3-*$(CAG)_{73}$ *nup60ΔKanMX* | This study |

| | | |
|---|---|---|
| SOY251 | *MAT***a** *ADE2 his3-11,15 lys2Δ trp1-1 ura3-1 RAD5 leu2-3,112::LacI-GFP-LEU2 Chr6int::lacop-lexAop-TRP1 HMG1-mCherry-HygMX his2-tRNA::URA3-(CAG)*$_{73}$ *nup1ΔCt::KanMX* | This study |
| SOY150 | *MAT***a** *ADE2 his3-11,15 lys2Δ trp1-1 ura3-1 RAD5 leu2-3,112::LacI-GFP-LEU2 Chr6int::lacop-lexAop-TRP1 HMG1-mCherry-HygMX his2-tRNA::URA3-(CTG)*$_{66}$ *nup84ΔKanMX* | This study |
| SOY141 | *MAT***a** *ADE2 his3-11,15 lys2Δ trp1-1 ura3-1 RAD5 leu2-3,112::LacI-GFP-LEU2 Chr6int::lacop-lexAop-TRP1 HMG1-mCherry-HygMX his2-tRNA::URA3-(CTG)*$_{67}$ *nup60ΔKanMX* | This study |
| SOY252 | *MAT***a** *ADE2 his3-11,15 lys2Δ trp1-1 ura3-1 RAD5 leu2-3,112::LacI-GFP-LEU2 Chr6int::lacop-lexAop-TRP1 HMG1-mCherry-HygMX his2-tRNA::URA3-(CTG)*$_{66}$ *nup1ΔCt::KanMX* | This study |
| SOY259 | *MAT***a** *ADE2 his3-11,15 lys2Δ trp1-1 ura3-1 RAD5 leu2-3,112::LacI-GFP-LEU2 Chr6int::lacop-lexAop-TRP1 HMG1-mCherry-HygMX his2-tRNA::URA3-(TGAAA)*$_{81}$ *nup84ΔKanMX* | This study |
| SOY257 | *MAT***a** *ADE2 his3-11,15 lys2Δ trp1-1 ura3-1 RAD5 leu2-3,112::LacI-GFP-LEU2 Chr6int::lacop-lexAop-TRP1 HMG1-mCherry-HygMX his2-tRNA::URA3-(TGAAA)*$_{81}$ *nup60ΔKanMX* | This study |
| SOY255 | *MAT***a** *ADE2 his3-11,15 lys2Δ trp1-1 ura3-1 RAD5 leu2-3,112::LacI-GFP-LEU2 Chr6int::lacop-lexAop-TRP1 HMG1-mCherry-HygMX his2-tRNA::URA3-(TGAAA)*$_{81}$ *nup1ΔCt::KanMX* | This study |
| SOY269 | *MAT***a** *ADE2 his3-11,15 lys2Δ trp1-1 ura3-1 RAD5 leu2-3,112::LacI-GFP-LEU2 Chr6int::lacop-lexAop-TRP1 HMG1-mCherry-HygMX his2-tRNA::URA3-(TTTCA)*$_{81}$ *nup84ΔKanMX* | This study |
| SOY276 | *MAT***a** *ADE2 his3-11,15 lys2Δ trp1-1 ura3-1 RAD5 leu2-3,112::LacI-GFP-LEU2 Chr6int::lacop-lexAop-TRP1 HMG1-mCherry-HygMX his2-tRNA::URA3-(TTTCA)*$_{81}$ *nup60ΔKanMX* | This study |
| SOY254 | *MAT***a** *ADE2 his3-11,15 lys2Δ trp1-1 ura3-1 RAD5 leu2-3,112::LacI-GFP-LEU2 Chr6int::lacop-lexAop-TRP1 HMG1-mCherry-HygMX his2-tRNA::URA3-(TTTCA)*$_{81}$ *nup1ΔCt::KanMX* | This study |
| SOY296 | *MAT***a** *ADE2 his3-11,15 lys2Δ trp1-1 ura3-1 RAD5 leu2-3,112::LacI-GFP-LEU2 Chr6int::lacop-lexAop-TRP1 HMG1-mCherry-HygMX his2-tRNA::URA3-(GAA)*$_{96}$ *nup84ΔKanMX* | This study |
| SOY253 | *MAT***a** *ADE2 his3-11,15 lys2Δ trp1-1 ura3-1 RAD5 leu2-3,112::LacI-GFP-LEU2 Chr6int::lacop-lexAop-TRP1 HMG1-mCherry-HygMX his2-tRNA:: URA3-(GAA)*$_{98}$ *nup60ΔKanMX* | This study |
| SOY256 | *MAT***a** *ADE2 his3-11,15 lys2Δ trp1-1 ura3-1 RAD5 leu2-3,112::LacI-GFP-LEU2 Chr6int::lacop-lexAop-TRP1 HMG1-mCherry-HygMX his2-tRNA:: URA3-(GAA)*$_{98}$ *nup1ΔCt::KanMX* | This study |
| DGY124 | *MAT***a** *leu2ΔEcoRI::URA3-HOcs::leu2ΔBstEII met15Δ0* | This study |
| SOY300 | *MAT***a** *leu2ΔEcoRI::URA3-HOcs::leu2ΔBstEII met15Δ0 nup1ΔCt::KanMX* | This study |
| SOY301 | *MAT***a** *leu2ΔEcoRI::URA3-HOcs::leu2ΔBstEII met15Δ0 nup84ΔKanMX* | This study |
| SOY311 | *MAT***a** *leu2ΔEcoRI::GAA*$_{100}$*-hphMX-HOcs::leu2ΔBstEII met15Δ0* | This study |
| SOY316 | *MAT***a** *leu2ΔEcoRI::GAA*$_{100}$*-hphMX-HOcs::leu2ΔBstEII met15Δ0 nup1ΔCt::KanMX* | This study |
| SOY313 | *MAT***a** *leu2ΔEcoRI::GAA*$_{100}$*-hphMX-HOcs::leu2ΔBstEII met15Δ0 nup84ΔKanMX* | This study |
| SOY312 | *MAT***a** *leu2ΔEcoRI::TTC*$_{100}$*-hphMX-HOcs::leu2ΔBstEII met15Δ0* | This study |
| SOY315 | *MAT***a** *leu2ΔEcoRI::TTC*$_{100}$*-hphMX-HOcs::leu2ΔBstEII met15Δ0 nup1ΔCt::KanMX* | This study |
| SOY314 | *MAT***a** *leu2ΔEcoRI::TTC*$_{100}$*-hphMX-HOcs::leu2ΔBstEII met15Δ0 nup84ΔKanMX* | This study |
| IKY287 | *MAT***a** *ADE2 can1-100 leu2-3,112 his3-11,15 ura3-1 trp1-1 LYS2 RAD5* | This study |
| IKY288 | *MATα DE2 can1-100 leu2-3,112 his3-11,15 ura3-1 trp1-1 LYS2 RAD5 mlp1ΔkanMX* | This study |

| | | |
|---|---|---|
| IKY289 | *MATα ADE2 can1-100 leu2-3,112 his3-11,15 ura3-1 trp1-1 LYS2 RAD5 mlp2ΔHIS3* | This study |
| IKY290 | *MAT**a** ADE2 can1-100 leu2-3,112 his3-11,15 ura3-1 trp1-1 LYS2 RAD5 mlp1ΔkanMX mlp2ΔHIS3* | This study |
| IKY291 | *MATα ADE2 can1-100 leu2-3,112 his3-11,15 ura3-1 trp1-1 LYS2 RAD5 nup1ΔCt::KanMX* | This study |
| IKY231 | *MAT**a** ADE2 can1-100 leu2-3,112 his3-11,15 ura3-1 trp1-1 LYS2 RAD5 nup2ΔKanMX* | This study |
| MKY12 | *MAT**a** ADE2 can1-100 leu2-3,112 his3-11,15 ura3-1 trp1-1 LYS2 RAD5 nup60ΔKanMX* | This study |
| MKY11 | *MATα ADE2 can1-100 leu2-3,112 his3-11,15 ura3-1 trp1-1 LYS2 RAD5 nup84ΔKanMX* | This study |
| FRY806 | *MATα ADE2 CAN1 his3-11,15 leu2-3,112 lys2Δ trp1-1 ura3-1 RAD5 hxt13ΔURA3 prb1ΔhphMX* | (Rosas Bringas et al., 2024) |
| SOY1 | *MATα ADE2 CAN1 his3-11,15 leu2-3,112 lys2Δ trp1-1 ura3-1 RAD5 hxt13ΔURA3 prb1ΔhphMX mlp1ΔkanMX* | This study |
| SOY2 | *MATα ADE2 CAN1 his3-11,15 leu2-3,112 lys2Δ trp1-1 ura3-1 RAD5 hxt13ΔURA3 prb1ΔhphMX mlp2ΔkanMX* | This study |
| SOY4 | *MATα ADE2 CAN1 his3-11,15 leu2-3,112 lys2Δ trp1-1 ura3-1 RAD5 hxt13ΔURA3 prb1ΔhphMX mlp1ΔkanMX mlp2ΔHIS3* | This study |
| SOY239 | *MATα ADE2 CAN1 his3-11,15 leu2-3,112 lys2Δ trp1-1 ura3-1 RAD5 hxt13ΔURA3 prb1ΔhphMX nup1ΔCt::kanMX* | This study |
| SOY279 | *MATα ADE2 CAN1 his3-11,15 leu2-3,112 lys2Δ trp1-1 ura3-1 RAD5 hxt13ΔURA3 prb1ΔhphMX nup2ΔkanMX* | This study |
| SOY5 | *MATα ADE2 CAN1 his3-11,15 leu2-3,112 lys2Δ trp1-1 ura3-1 RAD5 hxt13ΔURA3 prb1ΔhphMX nup60ΔkanMX* | This study |
| SOY6 | *MATα ADE2 CAN1 his3-11,15 leu2-3,112 lys2Δ trp1-1 ura3-1 RAD5 hxt13ΔURA3 prb1ΔhphMX nup84ΔkanMX* | This study |
| SOY293 | *MAT**a** ChromIII URA3-GAA$_{121}$-TetR395-URA-TRP1 ADE2 can1-100 his3-11,15 leu2-3,112 ura3-1 trp1-1 RAD5* | This study |
| SOY304 | *MAT**a** ChromIII URA3-GAA$_{121}$-TetR395-URA-TRP1 ADE2 can1-100 his3-11,15 leu2-3,112 ura3-1 trp1-1 RAD5 nup1ΔCt::KanMX* | This study |
| SOY307 | *MAT**a** ChromIII URA3-GAA$_{121}$-TetR395-URA-TRP1 ADE2 can1-100 his3-11,15 leu2-3,112 ura3-1 trp1-1 RAD5 nup60ΔKanMX* | This study |
| SOY308 | *MAT**a** ChromIII URA3-GAA$_{121}$-TetR395-URA-TRP1 ADE2 can1-100 his3-11,15 leu2-3,112 ura3-1 trp1-1 RAD5 nup84ΔKanMX* | This study |
| SOY302 | *MAT**a** ChromIII URA3-GAA$_{121}$-TetR395-URA-TRP1 ADE2 can1-100 his3-11,15 leu2-3,112 ura3-1 trp1-1 RAD5 rad27ΔKanMX* | This study |
| SOY306 | *MAT**a** ChromIII URA3-GAA$_{121}$-TetR395-URA-TRP1 ADE2 can1-100 his3-11,15 leu2-3,112 ura3-1 trp1-1 RAD5 rev1ΔKanMX* | This study |
| SOY50 | *MAT**a** ChromIII URA3- GAA$_{100}$-TetR269-URA-TRP1 ADE2 can1-100 his3-11,15 leu2-3,112 ura3-1 trp1-1 RAD5* | This study |
| SOY274 | *MAT**a** ChromIII URA3- GAA$_{100}$-TetR269-URA-TRP1 ADE2 can1-100 his3-11,15 leu2-3,112 ura3-1 trp1-1 RAD5 nup1ΔCt::KanMX* | This study |
| SOY310 | *MAT**a** ChromIII URA3-GAA$_{100}$-TetR269-URA-TRP1 ADE2 can1-100 his3-11,15 leu2-3,112 ura3-1 trp1-1 RAD5 nup60ΔKanMX* | This study |
| SOY213 | *MAT**a** ChromIII URA3- GAA$_{100}$-TetR269-URA-TRP1 ADE2 can1-100 his3-11,15 leu2-3,112 ura3-1 trp1-1 RAD5 nup84ΔKanMX* | This study |
| SOY303 | *MAT**a** ChromIII URA3- GAA$_{100}$-TetR269-URA-TRP1 ADE2 can1-100 his3-11,15 leu2-3,112 ura3-1 trp1-1 RAD5 rad27ΔKanMX* | This study |
| SOY326 | *MAT**a** URA3-GAA$_{100}$-TetR269-URA-TRP1 ADE2 can1-100 his3-11,15 leu2-3,112 ura3-1 trp1-1 RAD5 tof1ΔKanMX* | This study |

Table S4. GCR rates for Figure 1B and C.

| Sequence | GCR rate | Standard error | Fold change relative to no repeats |
|---|---|---|---|
| No repeats | $5.0 \times 10^{-10}$ | $5.9 \times 10^{-11}$ | 1 |
| $(CAG)_{101}$ | $1.8 \times 10^{-8}$ | $6 \times 10^{-9}$ | 3 |
| $(CAG)_{121}$ | $2.8 \times 10^{-8}$ | $2.5 \times 10^{-10}$ | 35 |
| $(CAG)_{161}$ | $9.2 \times 10^{-8}$ | $2.3 \times 10^{-8}$ | 55 |
| $(CTG)_{81}$ | $3.0 \times 10^{-9}$ | $6.3 \times 10^{-10}$ | 6 |
| $(GAA)_{81}$ | $2.7 \times 10^{-8}$ | $7.4 \times 10^{-9}$ | 54 |
| $(GAA)_{160}$ | $2.3 \times 10^{-7}$ | $8.8 \times 10^{-9}$ | 455 |
| $(TTC)_{81}$ | $9.0 \times 10^{-9}$ | $1.5 \times 10^{-9}$ | 18 |
| $(TTC)_{160}$ | $7.4 \times 10^{-8}$ | $5.9 \times 10^{-9}$ | 147 |
| $(TGAAA)_{51}$ | $9.2 \times 10^{-9}$ | $7.1 \times 10^{-11}$ | 1 |
| $(TGAAA)_{61}$ | $3.2 \times 10^{-9}$ | $1.8 \times 10^{-9}$ | 3 |
| $(TGAAA)_{81}$ | $8.6 \times 10^{-9}$ | $2.2 \times 10^{-9}$ | 9 |
| $(TGAAA)_{161}$ | $1.1 \times 10^{-7}$ | $3.7 \times 10^{-8}$ | 221 |
| $(TTTCA)_{51}$ | $1.2 \times 10^{-9}$ | $2.0 \times 10^{-11}$ | 1 |
| $(TTTCA)_{61}$ | $1.5 \times 10^{-9}$ | $2.8 \times 10^{-11}$ | 2 |
| $(TTTCA)_{81}$ | $4.6 \times 10^{-9}$ | $3.6 \times 10^{-10}$ | 5 |
| $(TTTCA)_{161}$ | $4.2 \times 10^{-8}$ | $6.9 \times 10^{-9}$ | 84 |

Table S5. Zoning analysis for Figures 2D and S1.

| Sequence | Cell cycle phase | Z1 # | Z1 % | Z2 # | Z2 % | Z3 # | Z3 % | Total number of cells | p-value relative to no repeats |
|---|---|---|---|---|---|---|---|---|---|
| No repeats | S/G2 | 49 | 32 | 53 | 35 | 49 | 32 | 151 | - |
| $(CAG)_{73}$ | S/G2 | 77 | 46 | 41 | 24 | 50 | 39 | 168 | 0.0099 |
| $(CTG)_{67}$ | S/G2 | 62 | 42 | 48 | 34 | 36 | 25 | 146 | 0.048 |
| $(TGAAA)_{81}$ | S/G2 | 95 | 42 | 64 | 28 | 67 | 30 | 226 | 0.038 |
| $(TTTCA)_{81}$ | S/G2 | 106 | 44 | 63 | 26 | 73 | 30 | 242 | 0.016 |
| $(GAA)_{99}$ | S/G2 | 68 | 49 | 39 | 28 | 32 | 23 | 139 | 0.0031 |
| $(TTC)_{180}$ | S/G2 | 78 | 46 | 44 | 26 | 46 | 27 | 168 | 0.0074 |
| No repeats | G1 | 45 | 34 | 49 | 37 | 39 | 29 | 133 | - |
| $(CAG)_{73}$ | G1 | 48 | 31 | 54 | 35 | 52 | 34 | 154 | 0.36 |
| $(CTG)_{67}$ | G1 | 61 | 37 | 60 | 36 | 44 | 27 | 165 | 0.33 |
| $(TGAAA)_{81}$ | G1 | 37 | 37 | 36 | 36 | 28 | 28 | 101 | 0.38 |
| $(TTTCA)_{81}$ | G1 | 46 | 38 | 39 | 33 | 35 | 29 | 120 | 0.27 |
| $(GAA)_{99}$ | G1 | 25 | 25 | 36 | 36 | 38 | 38 | 99 | 0.10 |
| $(TTC)_{180}$ | G1 | 38 | 31 | 50 | 41 | 34 | 28 | 122 | 0.37 |

P-values were calculated using Fisher's exact test.

Table S6. Colocalization analysis of STRs and the NPC for Figure 2E.

| Sequence | Colocalization # | Colocalization % | No colocalization # | No colocalization % | Total number of cells | p-value relative to no repeats |
|---|---|---|---|---|---|---|
| No repeats | 52 | 25 | 154 | 75 | 206 | - |
| $(CAG)_{68}$ | 74 | 38 | 123 | 62 | 197 | 0.0052 |
| $(CTG)_{63}$ | 59 | 42 | 80 | 58 | 139 | $6.3 \times 10^{-4}$ |
| $(TGAAA)_{82}$ | 69 | 54 | 59 | 46 | 128 | $1.2 \times 10^{-7}$ |

| | | | | | | |
|---|---|---|---|---|---|---|
| $(TTTCA)_{81}$ | 85 | 44 | 110 | 56 | 195 | $7.9 \times 10^{-5}$ |
| $(GAA)_{160}$ | 64 | 41 | 94 | 59 | 158 | 0.0014 |
| $(TTC)_{120}$ | 65 | 43 | 86 | 57 | 151 | $3.1 \times 10^{-4}$ |

P-values were calculated using Fisher's exact test.

## Table S7. Zoning analysis for Figure 2F.

| Treatment | STR | Phase | Z1 # | Z1 % | Z2 # | Z2 % | Z3 # | Z3 % | Total number of cells | p-value |
|---|---|---|---|---|---|---|---|---|---|---|
| No treatment | $(GAA)_{99}$ | S/G2 | 68 | 49 | 39 | 28 | 32 | 23 | 139 | - |
| 1,6-hexanediol | $(GAA)_{99}$ | S/G2 | 28 | 32 | 29 | 33 | 30 | 34 | 87 | 0.0094 |
| 2,5-hexanediol | $(GAA)_{99}$ | S/G2 | 37 | 47 | 23 | 29 | 18 | 23 | 78 | 0.47 |

P-values were calculated using Fisher's exact test.

## Table S8. Zoning analysis for Figure 3C.

| Mutant | STR | Phase | Z1 # | Z1 % | Z2 # | Z2 % | Z3 # | Z3 % | Total cells | p-value (compared to WT) |
|---|---|---|---|---|---|---|---|---|---|---|
| Wild type | No repeats | S/G2 | 49 | 32 | 53 | 35 | 49 | 32 | 151 | - |
| *nup84Δ* | No repeats | S/G2 | 65 | 34 | 65 | 34 | 60 | 32 | 190 | 0.41 |
| *nup60Δ* | No repeats | S/G2 | 48 | 34 | 42 | 30 | 50 | 36 | 140 | 0.42 |
| *nup1ΔCt* | No repeats | S/G2 | 49 | 29 | 60 | 36 | 58 | 35 | 167 | 0.32 |
| Wild type | $(CAG)_{73}$ | S/G2 | 77 | 46 | 41 | 24 | 50 | 39 | 168 | - |
| *nup84Δ* | $(CAG)_{73}$ | S/G2 | 58 | 34 | 59 | 34 | 56 | 32 | 173 | 0.013 |
| *nup60Δ* | $(CAG)_{73}$ | S/G2 | 74 | 46 | 53 | 33 | 33 | 21 | 160 | 0.51 |
| *nup1ΔCt* | $(CAG)_{73}$ | S/G2 | 73 | 34 | 85 | 40 | 54 | 25 | 212 | 0.016 |
| Wild type | $(CTG)_{67}$ | S/G2 | 62 | 42 | 48 | 34 | 36 | 25 | 146 | - |
| *nup84Δ* | $(CTG)_{67}$ | S/G2 | 55 | 33 | 61 | 36 | 53 | 31 | 169 | 0.045 |
| *nup60Δ* | $(CTG)_{67}$ | S/G2 | 62 | 40 | 42 | 27 | 51 | 33 | 155 | 0.38 |
| *nup1ΔCt* | $(CTG)_{67}$ | S/G2 | 73 | 34 | 75 | 35 | 64 | 30 | 212 | 0.077 |
| Wild type | $(TGAAA)_{81}$ | S/G2 | 95 | 42 | 64 | 28 | 67 | 30 | 226 | - |
| *nup84Δ* | $(TGAAA)_{81}$ | S/G2 | 53 | 36 | 50 | 34 | 43 | 29 | 146 | 0.16 |
| *nup60Δ* | $(TGAAA)_{81}$ | S/G2 | 89 | 43 | 55 | 26 | 64 | 31 | 208 | 0.48 |
| *nup1ΔCt* | $(TGAAA)_{81}$ | S/G2 | 50 | 36 | 38 | 28 | 49 | 36 | 137 | 0.18 |
| Wild type | $(TTTCA)_{81}$ | S/G2 | 106 | 44 | 63 | 26 | 73 | 30 | 242 | - |
| *nup84Δ* | $(TTTCA)_{81}$ | S/G2 | 62 | 37 | 60 | 36 | 46 | 27 | 168 | 0.098 |
| *nup60Δ* | $(TTTCA)_{81}$ | S/G2 | 70 | 43 | 59 | 37 | 32 | 20 | 161 | 0.52 |
| *nup1ΔCt* | $(TTTCA)_{81}$ | S/G2 | 54 | 37 | 46 | 32 | 45 | 31 | 145 | 0.12 |
| Wild type | $(GAA)_{99}$ | S/G2 | 68 | 49 | 39 | 28 | 32 | 23 | 139 | - |
| *nup84Δ* | $(GAA)_{99}$ | S/G2 | 27 | 36 | 31 | 41 | 17 | 23 | 75 | 0.047 |
| *nup60Δ* | $(GAA)_{99}$ | S/G2 | 70 | 53 | 36 | 27 | 26 | 20 | 132 | 0.29 |
| *nup1ΔCt* | $(GAA)_{99}$ | S/G2 | 56 | 37 | 40 | 26 | 57 | 37 | 153 | 0.022 |

Wild-type values are same as in Table S5. P-values were calculated using Fisher's exact test.

Table S9. GCR rates for Figure 5B.

| Genotype | Sequence | GCR rate | Standard error | Fold change relative to wild type | p-value (compared to WT) |
|---|---|---|---|---|---|
| Wild type | No repeats | $1.6 \times 10^{-9}$ | $3.2 \times 10^{-10}$ | 1 | - |
| *mlp1Δ* | No repeats | $2.0 \times 10^{-9}$ | $4.0 \times 10^{-11}$ | 1 | 0.033 |
| *mlp2Δ* | No repeats | $1.5 \times 10^{-9}$ | $2.2 \times 10^{-10}$ | 1 | 0.21 |
| *mlp1Δ mlp2Δ* | No repeats | $2.2 \times 10^{-8}$ | $5.5 \times 10^{-9}$ | 14 | <0.0001 |
| *nup1ΔCt* | No repeats | $6.4 \times 10^{-9}$ | $2.6 \times 10^{-9}$ | 4 | <0.0001 |
| *nup2Δ* | No repeats | $1.6 \times 10^{-9}$ | $5.0 \times 10^{-10}$ | 1 | 0.0293 |
| *nup60Δ* | No repeats | $8.3 \times 10^{-8}$ | $1.3 \times 10^{-8}$ | 50 | <0.0001 |
| *nup84Δ* | No repeats | $6.8 \times 10^{-8}$ | $2.2 \times 10^{-8}$ | 41 | <0.0001 |

P-values were calculated using Mann-Whitney U test.

Table S10. $(GAA)_{118}$ contraction rates for Figure 5C.

| Genotype | Sequence | Contraction rate | Standard error | Fold change to relative to WT | p-value (compared to WT) |
|---|---|---|---|---|---|
| Wild type | $(GAA)_{118}$ | $2.1 \times 10^{-4}$ | $3.8 \times 10^{-5}$ | - | - |
| *nup1ΔCt* | $(GAA)_{118}$ | $6.8 \times 10^{-4}$ | $1.2 \times 10^{-4}$ | 3 | 0.0001 |
| *nup60Δ* | $(GAA)_{118}$ | $2.9 \times 10^{-3}$ | $6.5 \times 10^{-4}$ | 13 | <0.0001 |
| *nup84Δ* | $(GAA)_{118}$ | $4.3 \times 10^{-3}$ | $1.1 \times 10^{-3}$ | 20 | <0.0001 |
| *rad27Δ* | $(GAA)_{118}$ | $1.9 \times 10^{-2}$ | $5.0 \times 10^{-3}$ | 87 | <0.0001 |
| *rev1Δ* | $(GAA)_{118}$ | $6.7 \times 10^{-4}$ | $1.3 \times 10^{-4}$ | 3 | 0.0005 |

P-values were calculated using Mann-Whitney U test.

Table S11. $(GAA)_{100}$ expansion rates for Figure 5D.

| Genotype | Sequence | Expansion rate | Standard error | Fold change relative to WT | p-value (compared to WT) |
|---|---|---|---|---|---|
| Wild type | $(GAA)_{100}$ | $2.1 \times 10^{-5}$ | $7.4 \times 10^{-6}$ | - | - |
| *nup1ΔCt* | $(GAA)_{100}$ | $2.1 \times 10^{-5}$ | $6.9 \times 10^{-6}$ | 1 | 0.88 |
| *nup60Δ* | $(GAA)_{100}$ | $1.7 \times 10^{-5}$ | $9.8 \times 10^{-6}$ | 0.8 | 0.70 |
| *nup84Δ* | $(GAA)_{100}$ | $5.01 \times 10^{-6}$ | $1.0 \times 10^{-6}$ | 0.2 | 0.0005 |
| *rad27Δ* | $(GAA)_{100}$ | $1.7 \times 10^{-3}$ | $2.2 \times 10^{-4}$ | 83 | 0.0001 |
| *tof1Δ* | $(GAA)_{100}$ | $1.0 \times 10^{-4}$ | $2.2 \times 10^{-5}$ | 5 | 0.0001 |

P-values were calculated using Mann-Whitney U test.

**References**


Aksenova, A.Y., P.W. Greenwell, M. Dominska, A.A. Shishkin, J.C. Kim, T.D. Petes, and S.M. Mirkin. 2013. Genome rearrangements caused by interstitial telomeric sequences in yeast. *Proc Natl Acad Sci U S A*. 110:19866-19871.

Casas-Delucchi, C.S., M. Daza-Martin, S.L. Williams, and G. Coster. 2022. The mechanism of replication stalling and recovery within repetitive DNA. *Nat Commun*. 13:3953.

de Bruin, D., Z. Zaman, R.A. Liberatore, and M. Ptashne. 2001. Telomere looping permits gene activation by a downstream UAS in yeast. *Nature*. 409:109-113.

Goldstein, A.L., and J.H. McCusker. 1999. Three new dominant drug resistance cassettes for gene disruption in Saccharomyces cerevisiae. *Yeast*. 15:1541-1553.

Rosas Bringas, F.R., Z. Yin, Y. Yao, J. Boudeman, S. Ollivaud, and M. Chang. 2024. Interstitial telomeric sequences promote gross chromosomal rearrangement via multiple mechanisms. *Proc Natl Acad Sci U S A*. 121:e2407314121.
